# A Mixed Reality–Based Telesupervised Ultrasound Education Platform on 5G Network Compared to Direct Supervision: Prospective Randomized Pilot Trial

**DOI:** 10.2196/63448

**Published:** 2025-06-12

**Authors:** Minha Kim, Meong Hi Son, Suhyeon Moon, Won Chul Cha, Ik Joon Jo, Hee Yoon

**Affiliations:** 1 Department of Emergency Medicine, Samsung Medical Center, Sungkyunkwan University School of Medicine, Seoul, Republic of Korea; 2 Department of Medical Sciences, Graduate School of Kangwon National University, Chuncheon, Republic of Korea; 3 Department of Digital Health, Samsung Advanced Institute for Health Science & Technology, Sungkyunkwan University, Seoul, Republic of Korea; 4 Data Innovation Center, Samsung Medical Center, Seoul, Republic of Korea; 5 Division of Biostatistics, Department of Academic Research, Kangbuk Samsung Hospital, Seoul, Republic of Korea

**Keywords:** ultrasonography, telemedicine, medical education, distance learning, fifth-generation network, mixed reality, ultrasound education, hospital, randomized pilot trial, pilot study, doctor, telesupervision, head-mounted display, primary outcomes, user experience, self-confidence, image quality, educational intervention, training experience, South Korea, telehealth

## Abstract

**Background:**

Ultrasound education is transitioning from in-person training to remote methods using mixed reality (MR) and 5G networks. Previous studies are mainly experimental, lacking randomized controlled trials in direct training scenarios.

**Objective:**

This study aimed to compare an MR-based telesupervised ultrasound education platform on private 5G networks with traditional in-person training for novice doctors.

**Methods:**

Conducted at a tertiary academic hospital from November to December 2023, the prospective unblinded randomized controlled pilot study assigned doctors without prior abdominal ultrasound education experience to either the telesupervision group (TG; n=20) or direct supervision group (DG; n=20). Participants received a 15-minute video lecture, conducted ultrasound on a phantom, and had 18 images scored by 2 blinded experts. Additionally, the TG received 5 minutes of training on the basic operation of a head-mounted display. Communication between doctors in the TG and supervisors was facilitated through a head-mounted display, whereas those in the DG interacted directly with supervisors. Primary outcomes were image quality scores, while secondary outcomes included procedure time, number of supervisor interventions, user experience using National Aeronautics and Space Administration-Task Load Index (NASA-TLX), System Usability Scale (SUS), and self-confidence through pre- and postsurveys.

**Results:**

Image quality scores and procedure times showed no significant differences between the groups (TG: 66.8 [SD 10.3] vs DG: 66.8 [SD 10.4], *P*=.84; TG: 23.8 [SD 8.0] min vs DG: 24.0 [SD 8.1] min, *P*=.95, respectively). However, the TG engaged in more educational interventions (TG: 4.0 [SD 2.5] vs DG: 0.8 [SD 1.1], *P*<.001), reflecting a more interactive training environment. TG participants reported lower NASA-TLX scores for mental demand (43.8 [SD 24.8] vs 60.6 [SD 22.4], *P*=.03), effort (43.1 [SD 22.9] vs 67.9 [SD 17], *P*<.001), and frustration (26.9 [SD 20.3] vs 45.2 [SD 27.8], *P*=.02), indicating a reduced cognitive load compared to the DG. The mean SUS score was also higher in the TG (66.6 [SD 9.1] vs 60.2 [SD 10.4], *P*=.046), suggesting better usability. Both groups showed significant improvements in confidence, with the TG showing notably greater improvement in abdominal ultrasound proficiency (pre-education TG: 1.6 [SD 0.9] vs DG: 1.7 [SD 0.9], *P*=.73; post-education TG: 3.8 [SD 0.9] vs DG: 2.8 [SD 1.0], *P*=.006).

**Conclusions:**

Although no significant differences in image quality scores were observed between groups, considerable differences in positive educational interactions, workload, and usability were evident. These findings emphasize the platform’s potential to enhance the ultrasound training experience, suggesting more interactive and efficient learning.

## Introduction

Ultrasounds, a low-cost, noninvasive, real-time imaging modality, have faced a paradigm shift in training methods due to the COVID-19 pandemic [1-6]. Ultrasonography is skill-intensive and operator-dependent, typically requiring hands-on practice with supervision [1]. However, technological advancements, the mismatch between global medical technology supply and demand, and social distancing measures due to infectious diseases have accelerated the transition of ultrasound education from traditional in-person training to digital training [7]. Some studies have shown no significant differences between ultrasound novices learning basic hands-on skills and basic ocular ultrasound skills through online self-learning compared to hands-on learning [8,9]. However, the real-time imaging characteristics of ultrasound necessitate real-time supervision during training [10]. Current tele-education methods, such as mobile phone apps, videoconferencing devices, and telemedicine software, have been used to connect learners with supervisors, enabling them to share and discuss ultrasound images [2,10]. Nevertheless, these methods present several issues. They transmit the ultrasound image but fail to show how the learner is holding the probe, thus hindering the supervisor’s ability to provide effective feedback on probe handling. Moreover, because the learner needs one hand to operate the probe, using the other to hold a communication device prevents effective manipulation of any additional materials or instruments.

To address these challenges, mixed reality (MR) systems have recently gained prominence in medical education [11-13]. MR involves merging physical objects with virtual information to facilitate interaction between the two [14]. Previous studies have explored the application of MR in contactless ultrasound education, demonstrating its potential to enhance the realism of educational experiences [15-17]. MR technology applies a virtual overlay to ultrasound images, allowing learners to study anatomy and pathology in a more immersive and interactive manner [18]. It also allows for immediate feedback and correction within a simulated clinical setting without requiring the supervisor to be physically present [19]. A key element of incorporating MR into ultrasound education is minimizing the delay in ultrasound image transmission between learners and supervisors. This can be achieved by using fifth-generation (5G) networks to ensure optimal transmission speeds [20]. 5G wireless networks reliably transmit data at speeds of up to 100 times faster than previous generations (10 gigabits per second), reducing latency to 1‐2 ms and multiplying simultaneous device connectivity by 100 [21]. The use of a 5G network is critical for minimizing latency during the transmission of ultrasound images, thereby enabling effective remote supervision.

Previous studies using MR systems and 5G networks have been mainly experimental, and there is a lack of randomized controlled trials evaluating the educational efficacy of these technologies in direct training scenarios [22]. Using MR on a 5G network, we aimed to assess the educational effectiveness of remote telesupervision with head-mounted display (HMD) versus traditional direct supervision, analyze the feasibility of the tele-education platform by surveying the workload and usability of novice sonographers, and address the prevalent educational challenges of ultrasound, which are marked by a scarcity of faculty, inadequate equipment, and limited training time. This study can be used as a reference to improve ultrasound training that emphasizes the tactile and manual skills essential to the discipline.

## Methods

### Study Design

This prospective randomized controlled pilot study was conducted at a tertiary academic hospital in South Korea from November 2023 to December 2023. The study aimed to compare an MR-based telesupervised ultrasound education platform using private 5G networks with traditional in-person training for novice doctors.

### Participants

We recruited medical doctors from the Samsung Medical Center interested in ultrasound education with supervision through online and offline bulletin boards. Participants had no prior experience with abdominal ultrasonography. To oversee the ultrasound examinations performed by each participant, the study engaged 2 faculty members from the emergency medicine (EM) department to provide supervision.

### Study Protocol

Overall, 40 participants were randomly assigned to the direct supervision group (DG) and telesupervision group (TG) using the lottery method. First, all participants completed a pre-survey and watched a 15-minute video lecture created using PowerPoint (Microsoft) for abdominal ultrasound education. The researcher provided an additional 5-minute training for the TG on basic HMD operations, including essential finger movements for HMD use, finger taps for navigating pages, and screen movement and resizing, allowing participants to adapt to the HMD while performing an ultrasound scan. Following the video lecture and HMD guidance, each participant was tasked with performing an abdominal ultrasound on the phantom and saving the 18 scanned images required. The DG were then provided with a printed handout, and the TG were provided with a PDF version of the handout presented as a hologram. During ultrasound scanning, the participants could ask questions and communicate with the faculty according to the assigned group’s supervision method. If the participants in either group needed assistance, they could seek help from their supervisors at any time. Next, the entire ultrasound procedure performed by the participants was recorded using a 360° camera. The time frame between the moment participants held the probe and the moment they returned the probe to the machine or pressed the “end exam” button to save images was measured. Finally, all participants completed a post-survey regarding their workload and usability.

### MR Telesupervised Ultrasound Education Platform

**Figure 1.**
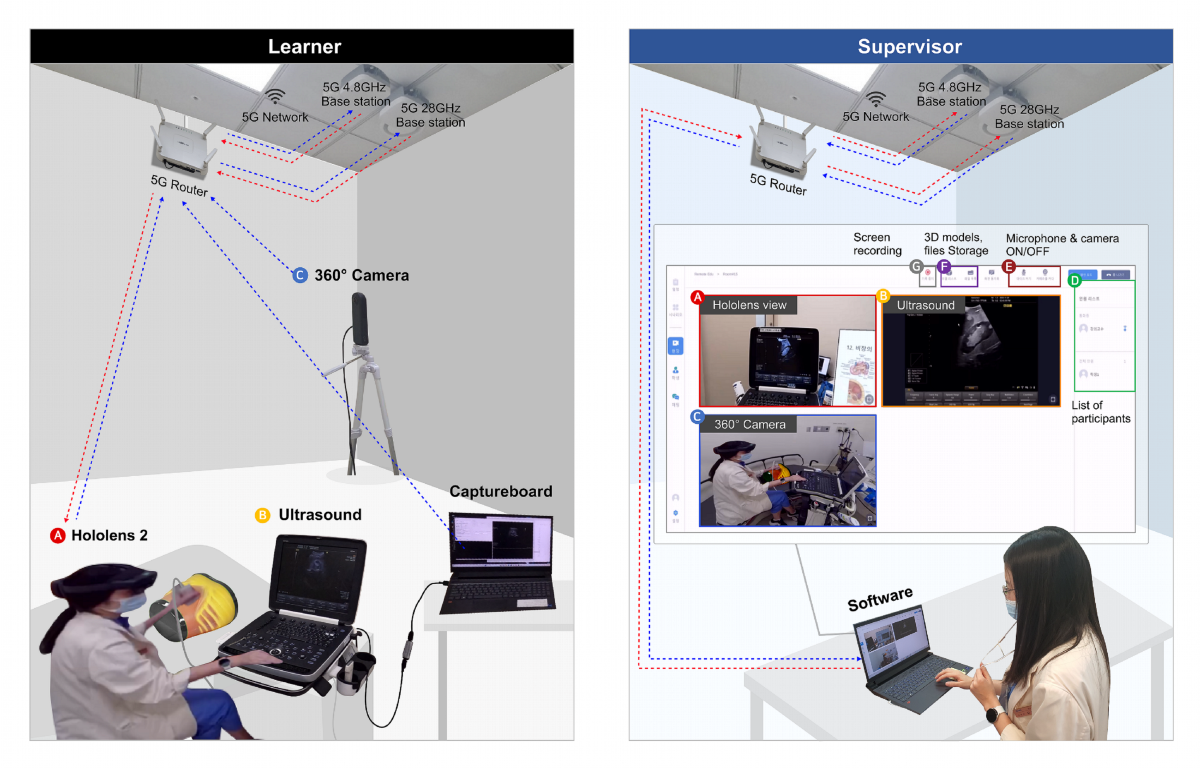
System architecture of MR telesupervised ultrasound education platform. The learner’s part consists of (A) an HMD, (B) an ultrasound device, and a laptop to capture ultrasound images through the capture board, and (C) a 360° camera. The software used by the supervisor displays the video streamed from (A), (B), and (C). Additionally, there is a (D) list of participants, (E) communication with the learner via microphone and camera that can be turned on and off by the supervisor, (F) the ability to send 3D objects, files, and images from the storage, and (G) a recording function. HMD: head-mounted display; MR: mixed reality.

The MR telesupervised ultrasound education platform consists of 3 components: the network, the supervisor (a laptop for supervision), and the learner (an ultrasound device, a laptop for capturing ultrasound images, an HMD, and a 360° camera) (Figure 1). The hospital installed 4.7 and 28 GHz 5G base stations, along with a private 5G core [23]. The 28 GHz band offers low latency and high speeds but has a limited range, making it suitable for hotspot use. Therefore, 4.7 GHz base stations were installed in multiple designated practice areas, including some operating rooms, wards, and emergency rooms, to provide wide-area coverage, while 28 GHz stations were used as hotspots. Download and upload throughput measured by iperf were 1453 and 147 Mbps, respectively.

The software used for telesupervision mainly consists of remote assistance services (facilitating MR communication between HMD users and supervisors), media services (media processing and streaming 360° camera, HMD, and ultrasound images), authentication (account authentication), image conversion, and 3-dimensional model services. Ultrasound images were converted to 3840×2160 NV12 at 30 frames per second via the capture board, converted to 640×480 on the laptop, and forwarded to the service platform via real-time streaming protocol. The HMD device used was a HoloLens 2 (Microsoft), which captures images at a resolution of 1088×612 and delivers them to the service platform as a web real-time communication media stream. The 360° camera used was a QooCam 8K (Kandao Technology Company Ltd), which captured a video at 4K (3840×2160) resolution at 30 frames per second and sent it to the service platform via the real-time streaming protocol.

Using this platform, the supervisor could simultaneously observe clear ultrasound images, the learner’s perspective via the HMD, and the learner’s ultrasound scan posture using a 360° camera. The supervisor manipulated the 360° camera image to observe the learner’s probe manipulation and offered assistance using visual and auditory cues through the HMD display. In the study hospital, communication latency from the HMD, ultrasound device, and 360° camera to the supervisor’s laptop was measured in 10 iterations, with mean values of 196 (SD 5) ms, 49 (SD 36) ms, and 797 (SD 26) ms, respectively.

### Abdominal Ultrasonography

The study materials were developed based on the abdominal ultrasound certification examination of the Korean Association of Clinical Ultrasound [24,25]. For novices, general ultrasonography information and knobology were introduced, and 18 abdominal ultrasound images were taught with the corresponding anatomical images: liver (7 images), biliary system (2), pancreas (2), spleen (1), kidney (4), and aorta (2) (Figure S1 in Multimedia Appendix 1). The ultrasound was performed using a Samsung Medison HM70A machine with a 1‐7 MHz curved array probe. Ultrasound settings were set by the researcher. Participants used an abdominal ultrasound phantom (ABDFAN, Kyoto Kagaku Co.). After the test, the 2 EM faculty members evaluated the images in a blinded manner. They assigned scores ranging from 1 to 5 to each image based on predefined evaluation criteria, and the mean score was used [26]. Missing images received a score of zero (Table S1 in Multimedia Appendix 2).

### User Experience and Confidence

To evaluate the feasibility of the MR-based telesupervised ultrasound education platform and its practical use, subjective user experience must be assessed. The National Aeronautics and Space Administration Task Load Index (NASA-TLX) is the most widely accepted and validated multidimensional tool that evaluates the overall workload across 6 dimensions: 3 dimensions measure the demands imposed on the subjects (mental demand, physical demand, and temporal demand), and 3 dimensions focus on how the subject deals with the task at hand (self-rated performance, effort, and frustration level). We used the raw NASA-TLX score, which is the arithmetic mean of all subscales, and did not weigh them [27,28].

The System Usability Scale (SUS) is a simple and widely adopted usability test tool based on questionnaires that can be used to evaluate software, mobile devices, and medical systems. The final scores were interpreted based on a well-established reference standard [29,30]. The SUS contains 10 questions based on a 5-point Likert scale. The odd questions were positive, and the even questions were negative. A higher SUS value indicates better product usability. Additionally, a pre- and post-training confidence survey was conducted using a 5-point Likert scale to assess the participants’ confidence in each organ and overall abdominal ultrasound before and after training [31,32].

### Evaluation of Outcomes

The primary outcome was the overall scan score of the 18 ultrasound images, with a total score of 90. Secondary outcomes included assessing the user experience through the NASA-TLX and SUS [29,33]. Additionally, we measured the time taken for the ultrasound procedure, the number of interventions made by supervisors, and self-confidence in performing abdominal ultrasound pre- and post-surveys.

### Statistical Analysis

All continuous variables were described as mean (SD), while categorical variables were described as numbers and percentages. For continuous variables, the analysis employed either the 2-sample *t* test or Wilcoxon rank-sum test, and for categorical variables, the *χ*^*2*^ test or Fisher exact test was used. Statistical significance was set at *P*<.05. All statistical analyses were conducted using R software (version 4.3.1; R Foundation for Statistical Computing).

### Ethical Considerations

The experimental protocol of the study involving human participants was carefully designed and implemented in strict compliance with the ethical standards of the Declaration of Helsinki. The study protocol was approved by the Institutional Review Board of the Samsung Medical Center (IRB file number 2023-10-015) and registered with ClinicalTrials.gov (NCT06171828). Each participant provided written informed consent before registration and received a small monetary compensation for their time and involvement in the study. All study data were deidentified to ensure privacy and confidentiality, in accordance with institutional guidelines. No identifiable images or personal details of individual participants are included in this manuscript.

## Results

### Demographics

**Figure 2.**
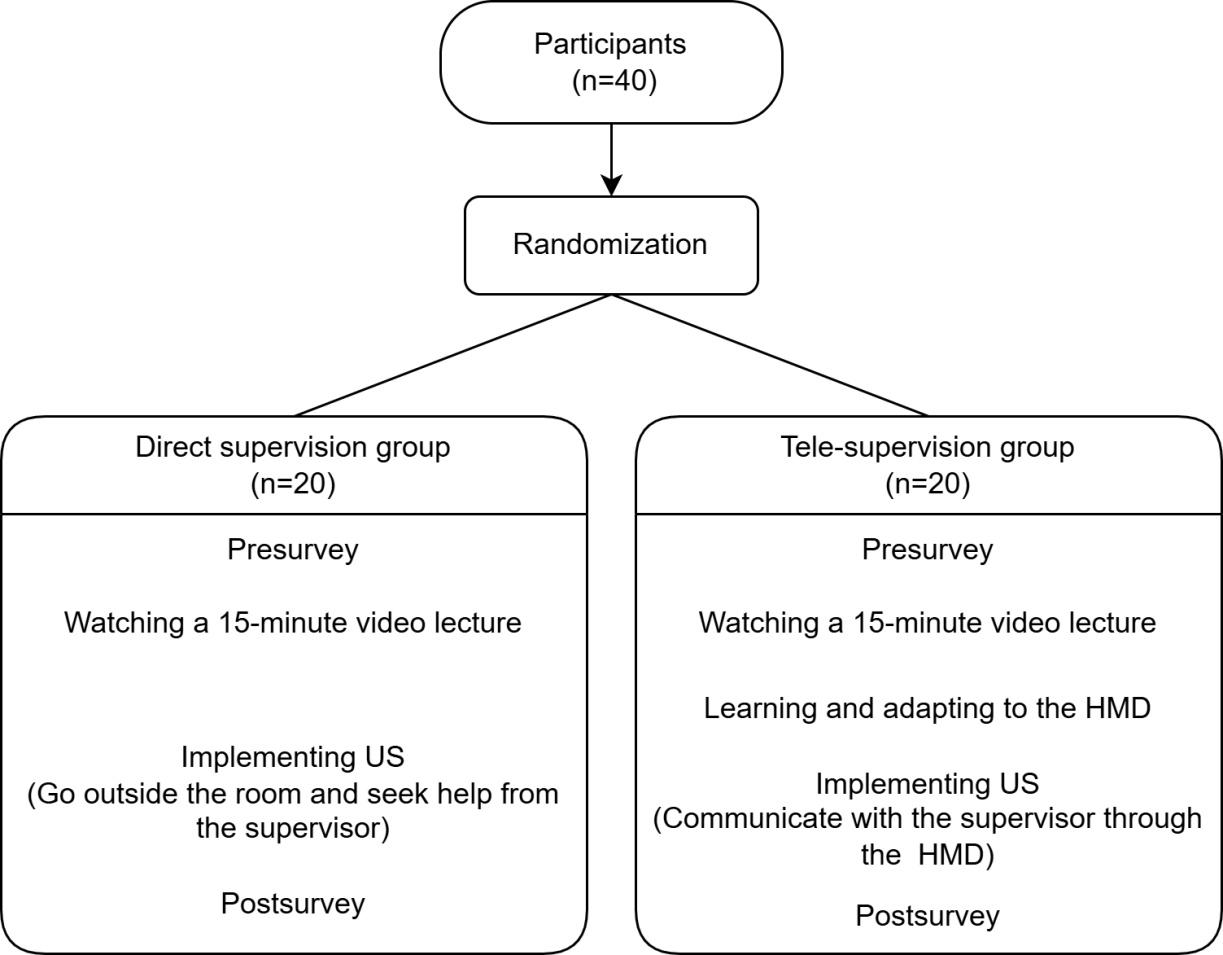
Flowchart of the study. HMD: head-mounted display; US: ultrasound.

From November to December 2023, 40 doctors who had not received prior training in abdominal ultrasonography participated in the study and completed the entire process, including subsequent surveys (Figure 2). The participants had a mean age of 28.3 years, and 25% (n=10) were men. The participants were comprised of interns (n=14, 35%), family medicine physicians (n=10, 25%), and pediatricians (n=6, 15%). No significant differences were observed in age, sex, physician grade, specialty, frequency of ultrasound (excluding abdominal ultrasound) use, or prior use of an HMD between the groups (Table 1).

**Table 1.** Demographic and basic characteristics of study participants.

Participant characteristics	Direct supervision group	Telesupervision group	*P* value
	(n=20)	(n=20)	
Age (years), mean (SD)	29.9 (3.9)	28.3 (3.5)	.19
Gender, n (%)			>.99
Men	5 (25)	5 (25)	
Women	15 (75)	15 (75)	
Physician-grade, n (%)			.57
Intern	6 (30)	8 (40)	
First-year resident	4 (20)	6 (30)	
Second-year resident	2 (10)	3 (15)	
Third-year resident	5 (25)	1 (5)	
Fourth-year resident	1 (5)	1 (5)	
Attending physician	2 (10)	1 (5)	
Specialty, n (%)			.27
Dermatology	1 (5)	0 (0%)	
Family medicine	7 (35)	3 (15)	
General surgery	1 (5)	1 (5)	
Internal medicine	0 (0)	2 (10)	
Intern	6 (30)	8 (40)	
Orthopedic surgery	0 (0)	2 (10)	
Pathology	0 (0)	1 (5)	
Pediatrics	3 (15)	3 (15)	
Radiology	2 (10)	0 (0)	
Frequency of ultrasound use, n (%)			.06
None	14 (70)	6 (30)	
1‐2 times per year	1 (5)	5 (25)	
1‐2 times per month	5 (25)	6 (30)	
1‐2 times per week	0 (0)	2 (10)	
More than 3 times per week	0 (0)	1 (5)	
Prior use of HMD^a^, n (%)	1 (5)	2 (10)	>.99

a HMD: head-mounted display.

### Overall Performance Between Groups

There were no significant differences in scores between the 2 groups (Table 2). The total scores for DG and TG were 67.5 (SD 10.4) and 66.8 (SD 10.4), respectively. The intraclass correlation coefficient between the scores given by the 2 EM faculty members was 0.9. The duration of the ultrasound between the 2 groups was not significantly different, with a mean time of 24.0 (SD 8.1) and 23.8 (SD 8.0) minutes for DG and TG, respectively. However, the number of interventions was significantly higher in the TG, with 4.0 (SD 2.5) interventions, compared to 0.8 (SD 1.1) in the DG.

**Table 2.** Analysis of abdominal ultrasound score between 2 groups^a^.

	Direct supervision group (n=20), mean (SD)	Telesupervision group, (n=20), mean (SD)	*P* value
Score			
Total (out of 90)	67.5 (10.4)	66.8 (10.4)	.84
Liver (out of 35)	24.7 (4.6)	24.6 (3.7)	.94
Gallbladder (out of 10)	7.5 (1.6)	8.2 (1.5)	.19
Pancreas (out of 10)	9.0 (1.0)	7.9 (2.2)	.06
Spleen (out of 5)	3.1 (1.5)	3.1 (1.5)	.96
Kidney (out of 20)	15.4 (2.8)	15.5 (2.7)	.86
Procedure time (min)	24.0 (8.1)	23.8 (8.0)	.95
Intervention	0.8 (1.1)	4.0 (2.5)	<.001

a The intraclass correlation coefficient between the scores given by 2 emergency medicine faculty members was 0.9.

### User Experience, Workload, and Confidence

All participants completed the NASA-TLX, SUS questions, and pre- and post-confidence surveys. The TG reported significantly lower scores in NASA-TLX for mental demand (mean 43.8 [SD 24.8] vs mean 60.6 [SD 22.4]), effort (mean 43.1 [SD 22.9] vs mean 67.9 [SD 17]), and frustration level (mean 26.9 [SD 20.3] vs mean 45.2 [SD 27.8]) compared to the DG, indicating reduced workload (Table 3).

**Table 3.** Analysis of National Aeronautics and Space Administration-Task Load Index (NASA-TLX) score between 2 groups.

Measure	Direct supervision group (n=20), mean (SD)	Telesupervision group (n=20), mean (SD)	*P* value
Mental demand	60.6 (22.4)	43.8 (24.8)	.03
Physical demand	40.1 (24.5)	33.1 (26.9)	.40
Temporal demand	47.6 (22.0)	36.9 (26.0)	.17
Self-rated performance	51.4 (22.5)	45.4 (26.3)	.44
Effort	67.9 (17)	43.1 (22.9)	<.001
Frustration level	45.2 (27.8)	26.9 (20.3)	.02

The mean SUS score of TG was significantly higher than that of DG (mean 66.6 [SD 9.1] vs mean 60.2 [SD 10.4]). Among all statements, “frequent use” (mean 4.3 [SD 0.6] vs mean 3.9 [SD 0.6]), “well-integrated system” (mean 4.1 [SD 0.6] vs mean 3.3 [SD 0.9]), and “learn quickly” (mean 4.2 [SD 0.7] vs mean 3.5 [SD 0.6]) were significantly higher in TG than in DG (Table 4).

**Table 4.** Analysis of standardized System Usability Scale (SUS) score between the 2 groups and each SUS statement^a^.

SUS questions (5-point Likert scale)	Direct supervision group (n=20), mean (SD)	Telesupervision group (n=20), mean (SD)	*P* value
I think that I would like to use this system frequently.	3.9 (0.6)	4.3 (0.6)	.02
I found the system unnecessarily complex.	2.1 (0.6)	2.0 (0.7)	.60
I thought the system was easy to use.	3.5 (0.6)	3.8 (0.6)	.13
I think that I would need the support of a technical person to be able to use this system.	3.5 (1.1)	4.1 (0.8)	.06
I found that the various functions in this system were well integrated.	3.3 (0.9)	4.1 (0.6)	.001
I thought there was too much inconsistency in this system.	1.9 (0.7)	1.7 (0.5)	.30
I would imagine that most people would learn to use this system very quickly.	3.6 (0.6)	4.2 (0.7)	.005
I found the system cumbersome to use.	2.1 (0.7)	2.3 (0.7)	.27
I felt very confident using the system.	3.3 (0.8)	3.7 (0.6)	.08
I needed to learn a lot of things before I could get going with this system.	3.8 (1.1)	3.3 (1.0)	.14
Total SUS score	60.2 (10.4)	66.6 (9.1)	.046

a Each statement has a 5-point Likert scale ranging from 1 (strongly disagree) to 5 (strongly agree).

**Figure 3.**
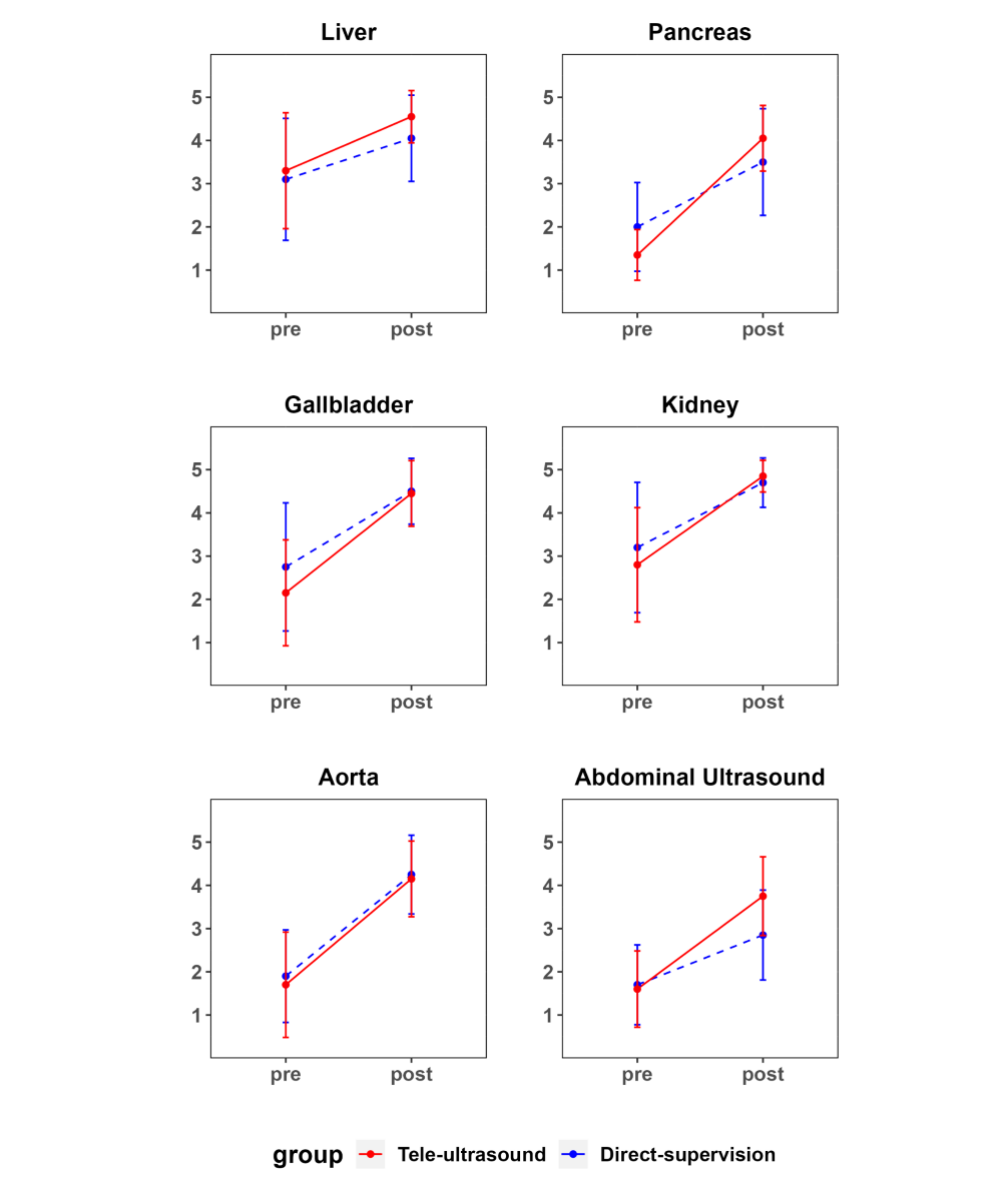
Pre-post assessment confidence levels by organ for each group. The confidence levels are displayed as mean values with standard deviations on a 5-point Likert scale.

The change in confidence for both groups showed significant increases in organ-specific measures from pre- to post-intervention (Table 5). Before the study, both groups generally had confidence levels below 3 points, with no significant differences except for the pancreas. After the study, most scores increased to the 4-point range, with the TG showing a significantly greater improvement, especially in abdominal ultrasound, referring to overall skill confidence in abdominal ultrasound (Figure 3).

**Table 5.** Confidence of each group before and after the study.

Confidence (5-likert scale)	Pre-education			Posteducation		
	Direct supervision group (n=20), mean (SD)	Telesupervision group (n=20), mean (SD)	*P* value	Direct supervision group (n=20), mean (SD)	Telesupervision group (n=20), mean (SD)	*P* value
Liver	3.1 (1.4)	3.3 (1.3)	.65	4.0 (1.0)	4.5 (0.6)	.07
Pancreas	2.0 (1.0)	1.4 (0.6)	.02	3.5 (1.2)	4.0 (0.8)	.10
Gallbladder	2.8 (1.5)	2.1 (1.2)	.17	4.5 (0.8)	4.4 (0.8)	.84
Kidney	3.2 (1.5)	2.8 (1.3)	.38	4.7 (0.6)	4.8 (0.4)	.33
Aorta	1.9 (1.1)	1.7 (1.2)	.59	4.2 (0.9)	4.2 (0.9)	.73
Abdominal ultrasound	1.7 (0.9)	1.6 (0.9)	.73	2.8 (1.0)	3.8 (0.9)	.006

## Discussion

### Principal Findings

This study compared an MR-based telesupervised ultrasound education platform on private 5G networks with traditional in-person training for novice doctors. Conducted through a randomized controlled trial with a relatively small sample size, the results indicated that the quality of abdominal ultrasound scans was consistent between the TG and the DG, indicating the platform’s ability to maintain educational integrity remotely. Additionally, telesupervision was associated with decreased mental demand, effort, and frustration, and showed positive impacts on usability and confidence levels. This study provides an important reference for advancing ultrasound training that focuses on the tactile and manual skills necessary for the field, even when using remote learning platforms.

Our findings affirm the feasibility, equivalence, and noninferiority of telehealth in ultrasound education, with outcomes comparable to in-person training [8,34-36]. Similar to other studies using HMD and MR guidance, telesupervision led to reduced cognitive loads [33]. Participants noted enhanced skill acquisition, easier command following, and greater confidence compared to traditional methods [37]. Challenges within the TG included the difficulty of managing dual tasks—performing ultrasound procedures while adapting to the HMD use. These issues could be distracting and potentially induce cybersickness, adversely affecting the learning experience [38]. As learners grow more familiar with HMD technology, these distractions are expected to decrease, improving the learning environment by minimizing psychological and physical barriers.

To address common technical issues like internet interruptions [39], delays, image freezes, and audio or video transmission problems [37], our study leveraged advanced 5G technology. This significantly mitigated latency issues and enhanced the potential for future applications of augmented reality in telehealth [40]. Unlike studies that reported complications with smartphone use during examinations [41], our implementation of an HMD freed the operator’s hands, enabling more realistic and immersive educational experiences.

In the study, several technical issues arose, including 2 instances of program crashes due to duplicate logins, 3 app crashes (with reconnection times within 5 s), and 1 microphone error, all of which required reconnection and extended the examination time for some in the TG group. Additionally, 2 delays in ultrasound image transmission and 1 hologram screen displacement caused by the HMD’s hand recognition required time adjustments but did not interrupt the session. Continuous updates to the system have been implemented to address these challenges and enhance the program’s stability, thus reducing the likelihood of similar disruptions in future studies.

Our system integrates an audiovisual cue system for real-time mentoring, addressing previous limitations related to the lack of physical guidance and verbal explanation difficulties [42]. The supervisor could use visual holographic cues such as arrows, lines, and images projected to the user’s view through an HMD, along with audio cues for precise probe manipulation. These cues were found to be the most effective means of conveying information to the operator [22]. However, it was challenging for supervisors to swiftly send holograms while engaging in conversation. Proficiency takes time for supervisors, and appropriate 3D objects need to be created and prepared.

To further enhance remote ultrasound education methods, our study emphasizes the importance of speed, stability, and security. In ultrasound training, the promptness of feedback is critical, making low latency essential. Maintaining latency under the 700 ms threshold is recommended for effective supervision [43-45]. Also, our platform includes 3 screens (HMD, ultrasound, and 360° camera), offering redundancy to maintain effective supervision even if one display encounters delays or freezes. This feature is essential for telemedicine, providing comprehensive observations from multiple angles. Furthermore, concerns about personal information leakage due to cyberattacks, particularly those targeting network and communication connections, are addressed by our system’s use of a 5G private network accessible only to registered devices [46,47]. This enhances security and protects sensitive data.

The developed platform’s versatility extends to various medical practices, including endoscopy and laparoscopy, using HDM interface for image transmission. With 5G base stations installed in hospital areas like operating rooms and emergency departments, it supports diverse skill training across medical fields. Its established infrastructure and innovative design facilitate a dynamic role swap between educators and learners, particularly enhancing surgical education by overcoming traditional constraints such as limited visual fields during surgery. Equipped with HMDs, surgeons can perform and broadcast procedures simultaneously, expanding learners’ perspectives and fostering interactive, immersive training experiences. This adaptability and functionality underscore the platform’s potential to revolutionize medical education, positioning it as a crucial tool for metaverse education platforms.

### Limitations

First, our study’s generalizability is limited by its single-institution setting within a private network, as technological and infrastructural conditions may differ across institutions. Additionally, this study did not include noninferiority testing due to the lack of a prior sample size calculation and statistical planning, which limits the robustness and generalizability of our findings. Future studies should address this aspect with appropriate sample sizes and statistical designs. Second, several technical challenges arose during the study, including program crashes, app errors, and ultrasound image transmission delays, which required additional time to resolve. While these issues were promptly addressed and did not affect the overall results or session flow, they highlight the need for further improvements in system stability to ensure consistent operation in future studies. Third, the lack of an assessment of the supervisor’s user experience creates a gap in the comprehensive evaluation of a platform’s overall effectiveness. Fourth, while a 5-minute HMD training session was provided, it may have been brief for participants with no prior HMD experience, potentially affecting initial ease of use. Although only a few HMD-related interventions were required, a longer training session could improve user familiarity and comfort in future studies (Table S2 in Multimedia Appendix 3). Fifth, the environmental conditions in the emergency department treatment room, with its inherent noise and commotion, might have affected the participants’ concentration and performance, adding an external variable to the study’s context. Finally, the use of a phantom model for simulation, although practical, may not fully capture the complexities of real patient interactions, potentially limiting the applicability of the training in actual clinical practice. Therefore, future studies should broaden participant bases, ensure technological robustness, evaluate all user experiences, and incorporate real patient scenarios to enhance the applicability of the findings and the platform’s utility in clinical education.

## Conclusions

This study demonstrated the potential of a novel MR-based ultrasound education platform using a 5G private network. Although no significant differences in scores were observed between groups, considerable differences in positive educational interactions, workload, and usability were evident. These findings emphasize the platform’s potential to significantly enhance the ultrasound training experience, suggesting a pivotal shift toward more interactive and efficient learning.

## Supplementary material

10.2196/63448 Multimedia Appendix 1 Standard images for abdominal ultrasound.

10.2196/63448 Multimedia Appendix 2 Evaluation criteria of ultrasonography.

10.2196/63448 Multimedia Appendix 3 Total number of interventions during experiment.

10.2196/63448 Checklist 1 Consolidated Standards of Reporting Trails (CONSORT) e-health checklist.

## Abbreviations

DG: direct supervision group
EM: emergency medicine
HMD: head-mounted display
MR: mixed reality
NASA-TLX: National Aeronautics and Space Administration Task Load Index
SUS: System Usability Scale
TG: telesupervision group
