# Correction: A Mixed Reality–Based Telesupervised Ultrasound Education Platform on 5G Network Compared to Direct Supervision: Prospective Randomized Pilot Trial

**DOI:** 10.2196/77586

**Published:** 2025-06-12

**Authors:** Minha Kim, Meong Hi Son, Suhyeon Moon, Won Chul Cha, Ik Joon Jo, Hee Yoon

**Affiliations:** 1 Department of Emergency Medicine, Samsung Medical Center, Sungkyunkwan University School of Medicine, 81, Irwon-ro, Gangnam-gu, Seoul, 06351, Republic of Korea, 82 0234102980; 2 Department of Medical Sciences, Graduate School of Kangwon National University, Chuncheon, Republic of Korea; 3 Department of Digital Health, Samsung Advanced Institute for Health Science & Technology, Sungkyunkwan University, Seoul, Republic of Korea; 4 Data Innovation Center, Samsung Medical Center, Seoul, Republic of Korea; 5 Division of Biostatistics, Department of Academic Research, Kangbuk Samsung Hospital, Seoul, Republic of Korea

In “A Mixed Reality–Based Telesupervised Ultrasound Education Platform on 5G Network Compared to Direct Supervision: Prospective Randomized Pilot Trial” (JMIR Serious Games 2025;13:e63448) the authors noted one error.

Affiliation 3 has been changed from:

Department of Health Sciences and Technology, Samsung Advanced Institute for Health Science & Technology, Sungkyunkwan University, Seoul, Republic of Korea

To appear as follows:

Department of Digital Health, Samsung Advanced Institute for Health Science & Technology, Sungkyunkwan University, Seoul, Republic of Korea

The correction will appear in the online version of the paper on the JMIR Publications website, together with the publication of this correction notice. Because this was made after submission to PubMed, PubMed Central, and other full-text repositories, the corrected article has also been resubmitted to those repositories.

