# Supplementary material for: A Mixed Reality–Based Telesupervised Ultrasound Education Platform on 5G Network Compared to Direct Supervision: Prospective Randomized Pilot Trial
Source: JMIR Serious Games. 2025 Jun 12;13:e63448. doi: 10.2196/63448 (PMC11788937; doi:10.2196/63448)

**Supplementary Figure 1.** Standard images for abdominal ultrasound. Overall, 18 images of a phantom are provided as a handout to participants. A. Longitudinal scan of the left lobe of the liver, B. Transverse scan of the left lobe of the liver, C. Subcostal scan of the confluence portion of the hepatic vein, D. Transverse scan of the right lobe of the liver, E. Intercostal scan of the right lobe of the liver, including the right portal vein, F. Longitudinal scan of the right hepatic lobe, including the right renal cortex, G. Intercostal scan of the liver dome, H. Longitudinal scan of the gallbladder, I. Long axial scan of the extrahepatic duct, J. Transverse scan of the pancreas, K. Transverse scan of the pancreas head, L. Longitudinal scan of the spleen, M. Longitudinal scan of the left kidney, N. Transverse scan of the left kidney, O. Longitudinal scan of the right kidney, P. Transverse scan of the right kidney, Q. Longitudinal scan of the aorta, R. Transverse scan of the aorta.


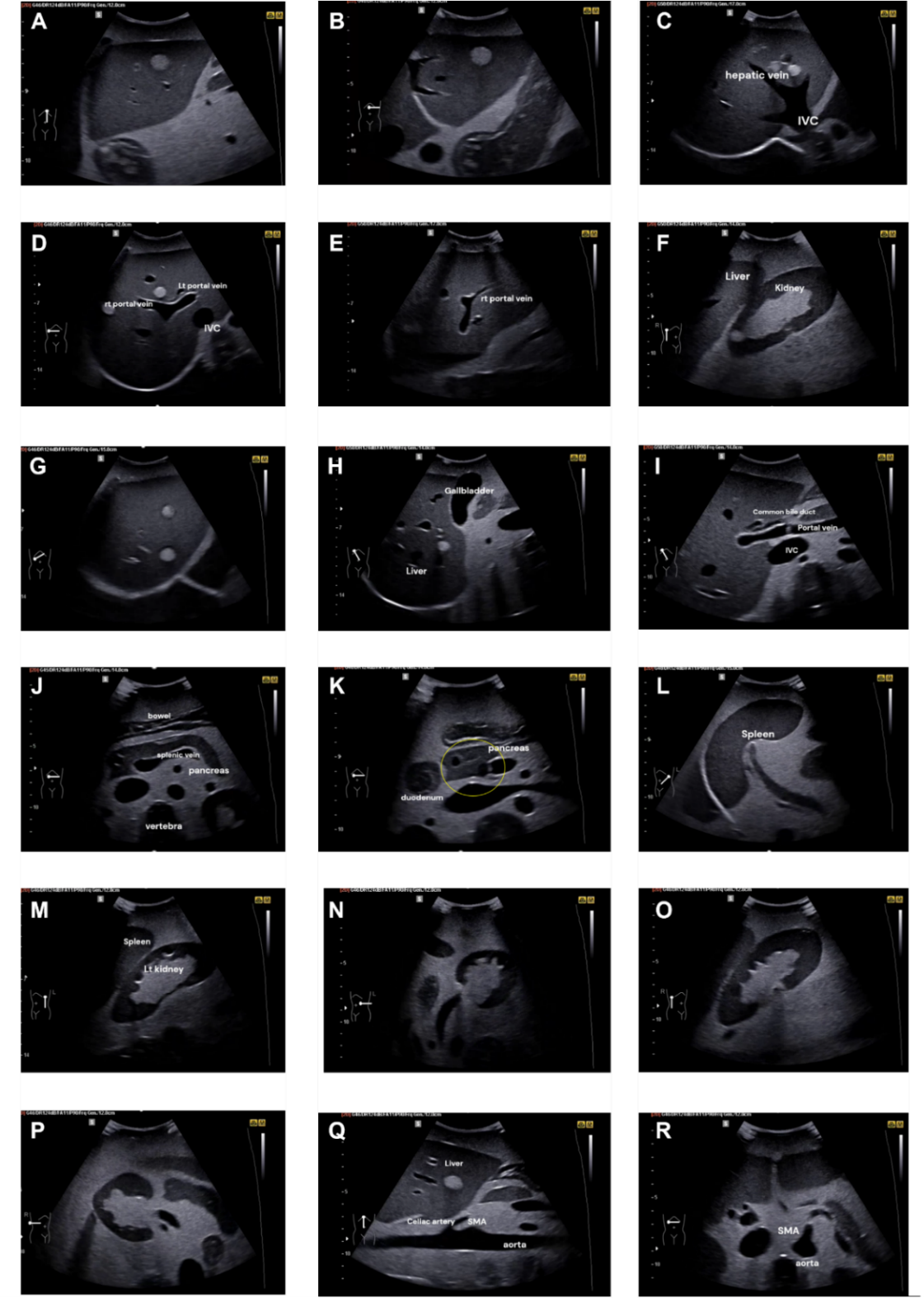

Supplement: Multimedia Appendix 1 [file games-v13-e63448-s001.docx]
