# Supplementary material for: A Mixed Reality–Based Telesupervised Ultrasound Education Platform on 5G Network Compared to Direct Supervision: Prospective Randomized Pilot Trial
Source: JMIR Serious Games. 2025 Jun 12;13:e63448. doi: 10.2196/63448 (PMC11788937; doi:10.2196/63448)
Supplement: Multimedia Appendix 2 [file games-v13-e63448-s002.docx]

**Supplementary Table 1.** Evaluation criteria of ultrasonography

| **Grading scale** | **Definition** |
| --- | --- |
| **1** | The target structure is completely unrecognizable. |
| **2** | The structure is minimally recognizable, but insufficient for scanning the target image. |
| **3** | The image meets the minimum criteria for scanning the target image, but it contains defects, including technical issues. |
| **4** | The image meets the minimum criteria necessary to scan the target image and has sufficient image quality to recognize the target structure. |
| **5** | The image meets the minimum criteria required to scan the target image, and all images are well captured. |
| Additional **-1** | Technical flaws: reversed marker orientation, completely inappropriate depth and gain settings, and so on. |
| Additional **-1** | The probe is not grounded across at least one-third of the screen. |
