# Supplementary material for: A Mixed Reality–Based Telesupervised Ultrasound Education Platform on 5G Network Compared to Direct Supervision: Prospective Randomized Pilot Trial
Source: JMIR Serious Games. 2025 Jun 12;13:e63448. doi: 10.2196/63448 (PMC11788937; doi:10.2196/63448)
Supplement: Multimedia Appendix 3 [file games-v13-e63448-s003.docx]

**Supplementary Table 2.** Total number of interventions during experiment.

| **Intervention type** | **Tele-supervision group** | **Direct supervision group** |
| --- | --- | --- |
| Organ localization | 8 | 0 |
| Probe adjustment | 60 | 11 |
| Ultrasound equipment usage | 4 | 4 |
| Basic ultrasound skills | 4 | 1 |
| HMD^a^ integrations | 4 | N/A^b^ |
| Total | 80 | 16 |

^a^HMD: Head-mounted display

^b^N/A: Not applicable
